# Speculating About Robot Moral Standing: On the Constitution of Social Robots as Objects of Governance

**DOI:** 10.3389/frobt.2021.769349

**Published:** 2021-12-02

**Authors:** Jesse De Pagter

**Affiliations:** Institute for Management Science, TU Wien, Vienna, Austria

**Keywords:** anticipatory governance, object of governance, robot ethics, robot governance, robot standing, speculative concept

## Abstract

In recent years, the governance of robotic technologies has become an important topic in policy-making contexts. The many potential applications and roles of robots in combination with steady advances in their uptake within society are expected to cause various unprecedented issues, which in many cases will increase the demand for new policy measures. One of the major issues is the way in which societies will address potential changes in the moral and legal status of autonomous social robots. Robot standing is an important concept that aims to understand and elaborate on such changes in robots’ status. This paper explores the concept of robot standing as a useful idea that can assist in the anticipatory governance of social robots. However, at the same time, the concept necessarily involves forms of speculative thinking, as it is anticipating a future that has not yet fully arrived. This paper elaborates on how such speculative engagement with the potential of technology represents an important point of discussion in the critical study of technology more generally. The paper then situates social robotics in the context of anticipatory technology governance by emphasizing the idea that robots are currently in the process of becoming constituted as objects of governance. Subsequently, it explains how specifically a speculative concept like robot standing can be of value in this process.

## Introduction

In recent years, the governance of robotic technologies has become an increasingly prominent issue within policy-making contexts. An important motivation behind the proclaimed need for such governance is that anticipatory approaches are crucial in order to keep pace with imminent transitions within society as the implementation of robots becomes increasingly widespread (Taeihagh, 2021). Such concerns over the insertion of robots into existing social contexts can at least partly be explained with reference to the widely diverging, speculative trajectories connected to the future of (social) robots (Suchman, 2019). These include predictions concerning the increasing applications and roles of (humanoid) social robots, which could potentially pose crucial challenges to the way social life has been organized for many years (Kim and Kim, 2013). Within the discussion on those challenges, the notion of robot standing is currently an increasingly important yet controversial concept. Complicating this discussion is the fact that in many cases, what needs to be governed - the widespread implementation of robots that could bring about fundamental societal transformations - has not yet been realized. While there are many signs and signals that such robots will or could soon be implemented on a broad scale, they are mostly currently still in investment and development stages (Mindell, 2015). Questions and debates regarding social life with robots therefore have quite a speculative character, and their relevance is sometimes questioned. The discussion on robot standing can be seen as an illustrative case of a controversy that is heavily vested in forms of speculative anticipation about the future of robots. This paper will take a closer look at the speculative character of the robot standing concept and discuss its usefulness for the process of constituting social robots as objects of governance.

The robot standing concept posits that artificial agents could have claims to novel forms of moral and/or legal status (Coeckelbergh, 2014). Thus, it is closely related to discussions on new understandings of technological artifacts and related changes in the conceptualization of agency. This paper does not provide new conceptualizations or ideas related to the discussion on robot standing itself; rather, it reflects on the usefulness of having such a discussion. That is to say, the speculative content of the robot standing concept is argued to be instrumental for the process of constituting robots as objects of governance. This process should be understood as open-ended: as (many types of) social robots are still emerging as technological artifacts of which the implementation has not yet fully materialized, so are the conceptual schemes that need to be developed to interpret and deal with the societal implications of those robots. The goal of this paper is first of all to provide new directions in the discussion of the significance and usefulness of speculative concepts like robot standing, by arguing that it can guide the development of ideas behind anticipatory robotic governance. In the context of fastly emerging robotics and AI, anticipatory governance is currently a prominent issue, as the main objective of such governance is to manage emerging technologies, while such management is still possible (Guston, 2014; Winfield and Jirotka, 2018) Second, in so doing, the paper is also meant to provide new arguments in response to opponents of the very idea of robot standing, who deem it irrelevant or harmful (e.g. Bryson, 2010; Birhane and van Dijk, 2020; Pasquale, 2020).

Therefore, while the debate on robot standing can be understood as an example of explicitly speculative engagement with emerging technology, this paper argues that speculative thinking on moral standing is an important and fruitful part of the process of robots becoming objects of governance. It does so via the following structure: The section below introduces important concepts underlying the notion of robot standing while summarizing the arguments of several voices in the debate. Based on this discussion, the section then examines the speculative elements inherent in the robot standing concept while also outlining the wider debate on the role of speculative concepts within the critical study of technology. The next section discusses what it means to understand robots as objects of governance. It explains how the process of constituting an object of governance should be understood, thereby elaborating on the role of speculative concepts like robot standing. The section after that discusses how the concept of robot standing itself can play a role in such a process when it comes to robotic governance. Finally, the conclusion will provide a short reflection on the role of philosophy of technology in the development of speculative concepts.

## Speculative Elements in the Discussion on Robot Standing

Concepts with speculative content can be helpful to anticipate technological potential. Nevertheless, the analysis of unrealized technological potential is an ambivalent topic in contemporary philosophy of technology as well as in other (social constructivist) fields that analyze the relationship between technology and society. In principle, the idea of social robots’ potential is rather straightforward, namely that the many different robotic technologies currently under development are accompanied by different expectations and promises regarding the future possibilities that those technologies present. However, as already indicated above, even though some new types of social robot technology might already be reality, many anticipated robots are still in the research and development process. At the same time, the public is teased with demonstrations of social robots, which are nevertheless largely still not part of daily social life. Autonomous social robots can therefore be understood to be in a phase where their sociotechnical potential is still mostly unrealized, while their implementation is simultaneously very much anticipated. Within the academic fields engaged in the critical study of technology, it is rather common to be very critical of such signs and signals of new futures. Moreover, conceptualizing the notion of future potential has proven to be difficult, especially when trying to abstain from determinist or instrumentalist views of technology, both of which are often seen as problematic (Wyatt, 2008; Dafoe, 2015). In fact, it is common practice in philosophy and the (qualitative) social sciences to analyze and often even debunk speculations regarding technological futures as a form of hubris. Technological potential, in such cases, is often implicitly or explicitly assumed to be conceptually problematic, theoretically incomprehensible, or denounced as a deterministic element in the discourse surrounding the technology under study (Heilbroner, 1994; Cressman, 2020). However, a possible way to engage with technological futures is to anticipate them by engaging with them while trying to analyze the ramifications of certain specific potentialities. I argue here that the debate on robot standing occupies an interesting position in this regard, as its engagement with the future potential of robotic technology contains elements that are explicitly speculative. As such, it is currently a relevant yet controversial concept that has already invited many different thinkers to engage with the possible consequences of robots as artificial agents.

Before delving into the topic of robot standing and its speculative character, it is useful to provide a short definition of what the notion of a “speculative concept” means in this context, especially since the term “speculative” has many different connotations. Speculative concepts, in this specific framework of emerging technology and its governance, can first of all be defined as concepts that aim to engage with the sociotechnical potential of an emerging technology. Sociotechnical potential in this case simply means that a multifaceted network of social and technical elements is considered during the assessment of that technology’s societal impact (Cressman, 2020). Furthermore, from this perspective, the sociotechnical potential of a specific technology is explicitly understood to be in a continuous state of controversy due to its undetermined character. Second, speculative concepts are understood to assist in the delineation of anticipatory scenarios based on actual developments. How realistic such anticipatory scenarios are, however, is always up for discussion, especially because engagement with the possible futures of technology already implies specific types of unknowns and contingencies. Third, the emphasis on speculative concepts as *concepts* is crucial. Concepts can be applied, discussed and reconceptualized in different contexts and can change their meaning depending on them. Finally, concepts are also different from overarching philosophical theories. Speculative concepts are in that regard smaller entities than theories. While they can certainly draw inspiration from larger philosophical frameworks, they are usually easier to apply in settings outside of these philosophical traditions.

### Introducing the Robot Standing Issue

From a broader philosophical point of view, the notion of robot standing and the arguments surrounding it can be seen as part of a general cultural fascination with machines as lively beings - a fascination which includes frequently mentioned historical examples such as Henri Maillardet’s automaton or Japanese karakuri puppets (Rossi et al., 2009). These examples of automata demonstrate how the notion of robots having a certain kind of standing, be it social, moral or otherwise, is part of the human fascination with alternative (non-human) forms of agency (Lindstrøm, 2015; Heffernan, 2019). However, this is not an easy topic, since objects, in whatever form, have been quite systematically barred from having any form of agency in modern societies (Harman, 2016). Generally, recent decades have seen rising interest in new forms of ontological pluralism and ethical extensionism, which pose novel ways of looking at objects in general and technological artifacts in particular (Chan, 2011; Pickering, 2017). As a part of this development, many different theories of non-human forms of agency have been developed. Bruno Latour, for instance, famously argued that modernity’s traditional subject-oriented moral theories conceal the agency and demands of non-human entities (Latour, 2005, 2014). In recent decades, several different academic fields, mainly in the social sciences and humanities, have either developed materialist critiques based on ideas of non-human agency, or have at least derived inspiration from those ideas (Law, 2008). Often, these theories and methods explicitly understand artifacts to carry forms of inherent sociality while emphasizing the (moral) agency of non-human entities like, for instance, technological artifacts (Gunkel, 2012). Many of those theories have speculative content or are based on concepts and ideas that are explicitly speculative in the sense that they refer to potential futures with new forms of agency. Others are based on entities that do not yet exist but can be anticipated. An important example of a speculative notion that is often mentioned in this context is Donna Haraway’s concept of the cyborg, which is developed to explore its emancipatory potential and unsettle solidified societal assumptions (Haraway, 1991).

Theorists like Latour and Haraway have conducted groundbreaking work on fundamentally novel ways of understanding and theorizing social agency. Although their theories and ideas do not explicitly engage with the topic of robot standing and its ramifications, an important discussion related to their endeavours is that of the human-machine boundary (Suchman, 2006). This discussion has become increasingly prominent in various academic fields during the last decade, as new developments in autonomous technology sparked an interest in exploring the implications and complications of such technologies (Floridi and Sanders, 2004; Dautenhahn, 2007). If they were to become reality on a wide scale, autonomous social robots are set to disturb modernist understandings of fundamental notions that are integral to the boundary between humans and machines, such as (moral) agency, responsibility, personhood, or empathy (Wallach and Allen, 2009). Several of those basic concepts are considered to be important to human identity and as such, have played a critical role in many (Western) legal, psychological and social concepts (Koops et al., 2013; Alač, 2016; Danaher, 2019; Fosch-Villaronga et al., 2020). If (social) robots were indeed to disturb such concepts, this could have profound implications for how humans understand themselves and how their societies are organized (Sætra, 2021). In that regard it is useful and important to think about the ethics of non-human entities (Gellers, 2020). For instance, synthetic persons, under which social robots would fall, present significant legal lacunae when it comes to most countries’ current legal systems (Bryson et al., 2017). Whereas most voices in this discussion would probably hesitate to ascribe proper sentience to robots, an important argument in the debate on standing is the discussion on the agentic appearance of social robots and the agency that should be attributed on the basis of that (Coeckelbergh, 2010; Nyholm, 2018). In this regard, the future potential of social robots becoming perceived as autonomous agents is generally an important topic in robotics research. There is already a lot of research in more applied fields like Human Robot Interaction (HRI) anticipating the agentic appearance of robots by applying so-called “Wizard of Oz studies”, in which robots’ autonomy and agency is imitated in order to conduct research about how humans would react to robots’ appearances and actions if they were to have agentic qualities (Maulsby et al., 1993; Riek, 2012). Closely related to this issue of appearance is the issue of control: Autonomous, agentic action by a machine assumes a certain lack of control by humans (Coeckelbergh, 2015; Wallach, 2015). This is also where important questions arise with respect to the governance of machine agency and the concepts of moral and legal standing attached to it. Hence, it is no surprise that (social) robots are being studied by legal philosophers and ethicists. Indeed, the regulation of robots, often in combination with artificial intelligence, has become an important topic in this field in recent years (Pagallo, 2013; Leenes et al., 2017; Turner, 2019). David Gunkel, an important proponent of the discussion on robot rights, nicely summarizes this by writing that “the question of robot rights (assuming that it is desirable to retain this particular vocabulary) makes a fundamental claim on ethics, requiring us to rethink the systems of moral considerability all the way down” (Gunkel, 2018a, 185).

Furthermore, the issue of robot standing has recently also started to become an actual topic in policy-making. An important example that is often mentioned in this context is the European Parliament (EP) considering the idea of electronic personality. This is not necessarily the same as robot standing, but certainly bears similarities in terms of its underlying dynamic. The EP’s report suggests the following with respect to the legal and economic notion of “electronic personality” (EP, 2017, §59f):

“creating a specific legal status for robots in the long run, so that at least the most sophisticated autonomous robots could be established as having the status of electronic persons responsible for making good any damage they may cause, and possibly applying electronic personality to cases where robots make autonomous decisions or otherwise interact with third parties independently”

In this quote, the EP argues that the actions and responsibilities of robots will render electronic personhood necessary in order to deal with their economic and legal consequences. Implicit in this understanding of such personhood is a notion of robot standing based on responsibility. It is exactly within such a context that attempts at anticipatory governance can be seen as guided by speculative concepts like robot standing. Nevertheless, this EP proposal immediately exposes the controversy of the issue, as it received serious backlash: an open letter was signed by 156 artificial-intelligence experts from 14 European countries, rejecting the EP’s recommendations (Nevejans, 2018). Thus, the fact that the autonomy of robots engaged in different types of social interactions could lead to significant challenges to the basic underpinnings of societal and legal understandings does certainly not mean that the participants in the debate are agreeing on robot standing. In fact, many consider the question of robot standing and the related idea of robot rights, very problematic. For instance, Joanna Bryson writes that there can be no real discussion about rights, since in the end robots are owned by humans (Bryson, 2010). Others call for a shift in focus towards safeguarding the welfare of all humans rather than focusing on robots while denouncing the issue of robot rights as something for AI and robotics futurists (Birhane and van Dijk, 2020; Pasquale, 2020). Furthermore, legal scholars have explicitly argued that robots should be deemed products, thereby excluding any considerations that understand robots as bearers of any rights or obligations (Bertolini and Aiello, 2018). Keeping this in mind, the goal of this paper is not necessarily to take a strong side in those debates, but much rather to explicitly consider the role of speculative content implicit in the robot standing concept and reflect on it as such. In order to do that, we must take a step back and be more explicit about the character of this speculative content, which will be done below.

### Robot Standing and Its Speculative Ethics

Whereas the arguments above demonstrate various ideas about robot standing, it is important to seriously consider whether the discussion as a whole is too far-fetched and excessively rooted in speculative, futuristic arguments that bear no ground in engineering reality. David Gunkel, who was already mentioned above, takes an important, quite distinctive voice in this debate, as he strongly argues for exploring “robot rights”, an issue closely connected to the topic of robot standing (Gunkel, 2018a; 2018b). In his book on robot rights, he explains and reviews the different positions on the question of robot standing. Gunkel quotes and refers to an array of philosophers who are mostly sceptical about the usefulness of the notion of robot rights, a notion that is closely related conceptually to robot standing. The main point in this view is that robot rights are “unthinkable”. Gunkel himself counters this criticism by arguing that it is a task of critical thinking to expose why the unthinkable is unthinkable, thereby “confronting and thinking the unthinkable” (2018a, 51). Furthermore, he argues that ethics is the field with the tools and obligations to ultimately challenge the status quo, which is exactly how moral theories and practices evolve. The task of ethics, he writes, is to “stress-test and question the limitations and exclusions of existing moral positions and modes of thinking. Defending orthodoxy is the purview of religion and ideology; critically testing hypotheses and remaining open to revising the way we think about the world in the face of new challenges and opportunities is the task of science” (2018a, 52).

Gunkel’s focus on the role of ethics is interesting here, as the field generally has a rather unique position when it comes to engagement with speculative technological futures. Much of the philosophical work focused on ethical thinking with regards to technological development is in fact participating in the anticipation of future social and legal ontologies. That is to say, ethicists who study robotics (or other emerging technologies, e.g. nanoethics) often actively engage with questions that are somewhat speculative in order to discuss ethical challenges and lacunae that the future of those technologies could bring about. One might think this only applies to posthumanist ethics, but this is certainly not the case. Many of the current discussions around social robots in philosophy are focused on describing and analysing new ontologies regarding the human-machine boundary. Accordingly, ethicists have extensively engaged in speculative explorations of future legal and social ontologies and their consequences for human social life with robots. Within philosophy, the examination of such questions and their potential implications has been a natural fit for several of its subdisciplines, presenting a great opportunity to gain practical and effective relevance in a society that is increasingly organized around expertise. Furthermore, this type of engagement has arguably increased ethicists’ interdisciplinary collaboration with many other fields in robotics, such as HRI, legal theorists, robot engineers and so on.

The question remains why such interdisciplinary ethics approaches based on speculative concepts are considered problematic. One of the staunchest critics of this speculative element in the ethics of emerging technology, Alfred Nordmann, provides clear insight into this issue. In his ethical and technophilosophical deliberations on the future of nanotechnology, Nordmann strongly argues against what he calls ”speculative nanoethics” which, he argues, is based on the technological hubris of “if-and-then” rhetorics (Nordmann, 2007). Nordmann, who refers to himself as a “reluctant ethicist”, problematizes various ethical approaches that are imaginative with respect to the future, exhibiting a clear preference for less imaginative approaches that “bring to light how less spectacular, more familiar technologies shape and reshape, perhaps transform social interactions, individual agency, and a sense of subjectivity or self” (p. 44). In a paper with Arie Rip, Nordmann writes that “worries about the most futuristic visions of nanotechnology can cast a shadow on all ongoing work in nanoscience and technology” (Nordmann and Rip, 2009, 274). By making these points, Nordmann started a fruitful and important discussion within the field of nanoethics, but also in the larger context of the critical analysis of anticipatory approaches (Nordmann, 2014). Various other works have since discussed arguments complementary to Nordmann’s. For instance, Ibo van de Poel proposes an alternative to speculative anticipatory approaches when he argues for the gradual experimental introduction of new technologies, while assessments regarding the acceptability of such introductions should be based on ethical frameworks (van de Poel, 2016). On the other hand, Nordmann’s arguments have also been strongly criticized. For example, Armin Grunwald argues that instead of ‘speculative ethics’, we should speak about ‘explorative philosophy’ which “must develop methods and procedures of assessing pictures of uncertain futures with respect to their degree of rationality” (Grunwald, 2010, 99). Cynthia Selin writes in a direct response to (Nordmann, 2014) article that “foresight practices are meant to contrast the techno-scientific, future-grasping hubris that has been under scrutiny from STS scholars (amongst others) for decades,” while also writing that Nordmann fails to systematically categorize what forms of speculation exactly are unacceptable (Selin, 2014, 103).

Whereas this discussion on the role of speculative concepts has mostly been confined to insiders within academic fields such as philosophy of technology and science and technology studies (STS), the notion of robot standing and its speculative character have caused a stir both inside and outside of academia. As such, it is a particularly good example of the role of speculative concepts in the analysis of (emerging) technology. In this context, it is interesting when David Gunkel writes that “science fiction is both a useful tool for and a significant obstacle to understanding what the term “robot” designates” (2018a, 18). Importantly, Gunkel emphasizes here the importance of understanding that what “robot” means is socially negotiated and that “word usage and terminological definitions shift along with expectations for, experience with, and use of the technology” (2018a, 23). Those quotes already provide an indication on how speculative concepts like robot standing can be useful from an anticipatory governance perspective. First of all, it is particularly challenging to engage in anticipatory governance that prepares for futures involving potentially disruptive technologies. While it has already been demonstrated that the development and application of speculative concepts is a contested practice in general, my goal is to further establish the development and implementation of specific kinds of speculative thinking *within* the empirical tradition of the critical study of technology. This research tradition has already provided very relevant insights for policy ideas while directly engaging with technology in the making via both philosophical and (qualitative) social science methods (see e.g. Boden et al., 2017; Bösl and Bode, 2018; AIHLEG, 2019). Robotic technologies represent a great example of this type of engagement since their societal impact is currently highly anticipated. Furthermore, as will be argued below, the concept of robot standing provides valuable insight into the way a speculative concept can be used in the (empirical) critical study of technology and its governance challenges. Even if one agrees with (some of) the problematizations concerning Nordmann’s so-called “if-and-then” rhetoric, the main point here is that it remains important to engage with the issue of future contingency in technological development and its governance through concepts like robot standing and the debates around it. It is exactly in such a context that the robot moral standing concept is explored in the following section.

## Robots as Objects of Future Governance

The main point of this section is to argue how a speculative concept like robot standing can be of value in the process of constituting robots as objects of governance. This process is explicitly understood to be far from completed, and the goal is to develop an argument that explores speculative thinking on moral standing as an important and worthwhile element of this process. It should be mentioned in this regard that in several policy areas, robots are already very much constituted as objects of governance. For instance, industrial robots have been used in industry for many years. In this context, policies regulating and governing robots are clearly established, such as in terms of safety and liability: for instance in the context of the EU, very specific rules apply when it comes to safety and industrial robots, regulated by policies such as the Machinery Directive (Directive 2006/42/EC), the Framework Directive for Occupational Safety and Health (Directive 89/391/EEC) and others, often depending on the context of use. In this case, robots are mostly defined (and thus also regulated) as being possibly dangerous to workers’ health and safety. Furthermore, robots have long been a part of policy discourse in strategic economic policy-making, in which their presence has unsurprisingly become an indicator of an economy’s rate of automation, innovation and economic progress. However, the main issue in the case of the discussion around robot autonomy is not how robots are currently defined as objects of governance in various policy-making areas, but rather how their potential future characteristics could render them objects of governance in policy areas where they were either not considered before or were considered in a different manner. This might even lead to the emergence of completely new policy areas. In that regard, it is important that robots be explicitly considered an emerging technology, as will be argued below.

### Governance of Emerging Technology and Its Difficulties

The governance of new technology is often based on the assumption that a technology is developed first, after which policy-making initiatives are created to govern its implementation in society so as to regulate certain uses of that technology. Even though many concepts and theoretical frameworks of technological development have argued against this assumption in different ways, it remains a rather stubborn notion. In addition, it can also be connected to a more fundamental problem regarding the character of governance versus the character of technological development. An often-cited and well-defined expression of this problem is the Collingridge dilemma, which still functions as an important reference in fields like responsible research and innovation (RRI) and technology assessment (TA) (Genus and Stirling, 2018). This dilemma was defined by David Collingridge in his 1980 book “The social control of technology,” with the book’s preface providing a concise and clear definition: “By the time undesirable consequences are discovered, however, the technology is often so much part of the whole economic and social fabric that its control is extremely difficult” (Collingridge, 1982, 11). Particularly in the current moment, which is characterized by technological changes that are changing socioeconomic and political realities in a rapid and profound manner, the dilemma of control is often felt to be particularly prevalent. Examples are multiple, but a prominent one has been the use of big data analytics on social media (e.g. for election campaigns). It is therefore not surprising that calls for a change of approach to technology governance are particularly strong at the moment (Bratton, 2015; OECD, 2017; Schwab, 2017; Winfield and Jirotka, 2018).

The governance of emerging technologies presents an important challenge that has been addressed in different ways in various social science and humanities disciplines. It has been repeatedly noted that the governance of emerging technologies can be seen as quite a specific type of governance (Kuhlmann et al., 2019; Ulnicane et al., 2021). Based on the discussion and analysis of different emerging technologies throughout the years, a useful body of literature has developed discussing the particular status of emerging technologies in policy-making. (Bonnin Roca et al., 2017; Dorbeck-Jung and Bowman, 2017; Kaebnick and Gusmano, 2018). First of all, as previously mentioned, emerging technologies often have the potential to *cause effects on a broad scale* in society (Rotolo et al., 2015). An important issue for the governance of emerging technologies like robotics is that initially relatively small-scale projects can have severe ramifications in the near future, not least because financing schemes in the startup economy render high-risk/high-reward ventures more likely (McNeill, 2016). When it comes to social robots specifically, the main issue concerns their increasing ability to participate in different parts of social life. As demonstrated in the section above, many philosophers have been discussing potential consequences for the organization of social life, and the robot standing debate can very much be seen as a part of this larger discussion. Second and related to the first point, policy-making developments regarding emerging technologies are generally characterized by *widely divergent expectations* concerning the potential futures of those technologies. Apart from general expectations, this also applies very much to sociotechnical imaginaries in policy-making, as has been repeatedly demonstrated (Kearnes et al., 2006; Vesnic-Alujevic et al., 2016; Rieder, 2018). An important reason for this is that emerging technologies are usually surrounded by hype and various buzzwords. In that sense, it is beneficial to apply some vocabulary from STS research, which has a good track record analyzing emerging technologies in relation to public attitudes and governance. A useful term here is “sociotechnical controversy” (Bonneuil et al., 2008). Central to the notion of sociotechnical controversies and their emergence is that they are continuously in the making and are subject to negotiation processes among different stakeholders. Fields like (global) governance studies and STS have extensively analyzed such processes. Finally and related to the first two points, there is often a strong *public interest* in the (potential) development of emerging technologies. This is an issue that is particularly prominent in the case of emerging technologies and their future trajectories, since emerging technologies are often characterized by many different expectations and speculations regarding their future development. Public attitudes towards the sociotechnical controversies around emerging technologies are therefore usually considered to play an important role in the uptake of these technologies. Autonomous technologies like robots in general and social robots more specifically are a particularly prominent issue in this respect. Their (potential) autonomy has been a recurring major theme in many different kinds of media and art for many years, while recent developments in AI technology could indeed bring about a strong leap in the actual autonomy of robotic devices.

### Emerging Technologies as Objects of Governance

Above it has become clear that robots, seen as an emerging technology, are to be understood as a challenge in terms of governance. Furthermore, when it comes to issues of governance, it is important to note that emerging technologies suffer from a particularly strong form of fuzziness about their status as objects of governance. This very much applies to emerging robotics (and AI) as well. Central to this problem are challenges regarding the contingencies when it comes to robots as objects of governance. Those contingencies can be understood in two different ways. The first concerns future contingency and is the most straightforward: uncertainty about future technological developments makes technology governance a difficult issue. We do not yet know the future of robots as objects of governance, but want to anticipate it in order to implement governance measures in a timely manner. The second concerns ontological contingencies regarding the object of governance itself. The question here relates to the phenomena that are considered to be part of robots, as well as the different ways those phenomena can be rendered governable. Robots are as such a particularly fuzzy and dynamic phenomenon that is difficult to fully grasp through the different policy-making instruments that are available or could potentially be developed in the future.

In both of these cases of fuzziness, speculative concepts can be instrumental in the constitution of objects of governance by rendering them more explicit. That is to say, by carefully developing arguments on the basis of speculative concepts such as robot standing, we render the (perceived) autonomy and agency of robots into explicit phenomena that define robots in their social context. Relevant here is how speculative concepts can influence the way in which emerging technologies become constituted as future objects of governance. I argue here that it is exactly the speculative element that can help in the further development of anticipatory robotics governance. In this way, the role of forecasting practices as well as policy instruments in general can evolve, especially when it comes to specific technological trends like the emergence of new types of robots. As demonstrated, for instance, by the European Parliament’s notion of electronic personality, this type of governance is experiencing continuous evolution as new policy ideas gradually develop.

As already explained above, when it comes to robotics, applied ethics fields like robot ethics have gained influence in policy-making discourse around emerging technologies in recent years. From a governance perspective, this can be seen as a way to anticipate future changes (Brey, 2012). The goal here is thus to develop a better understanding of how technologies like robots become constituted as objects of governance and subsequently elaborate how approaches to future contingencies in the governance of technology are materialized during this process. This will be instrumental for the subsequent discussion section, which further elaborates how robot standing and its speculative content can play a role in the anticipation of autonomous social robots. The analysis of policy-making efforts around unpredictable issues with a high level of controversy and a strong presence of buzzwords has developed considerably in recent decades, especially in STS research (Fortun, 2001; Hilgartner, 2009). In that regard, it is useful to elaborate on robot governance by drawing upon literature from this field and other policy research around the notion of “objects of governance”. Other terms that are often used in this context are “governance object” or “object of government” (Lezaun, 2006). When used as a concept for analysis, an important assumption is that governance arrangements around objects of governance can be traced back to contested representations in earlier phases of their emergence as objects of governance (Allan, 2017). The underlying idea is that objects of governance are hybrid, co-produced entities that emerge from complex interactions between expert knowledge, political interventions and mundane practices (Allan, 2018). In other fields of research, it has already been demonstrated how epistemic communities play a central role in the development of new and altered policy ideas (Swinkels, 2020). Examples of such research are: the climate as an object of (global) governance (Bulkeley, 2005; Allan, 2017), urban warming as an object of (local) governance (Boezeman and Kooij, 2015), or creative thinking as an object of governance and geopolitical concern in the United States military context during the Cold War (Van Eekelen, 2017). As such studies show, anything can become a governance object as long as it becomes distinguishable and is rendered governable. Bentley Allan provides a comprehensive description of governance objects when he defines them as “concatenations of knowledges, artifacts, physical phenomena, and practices that have been yoked together and constituted as an entity distinct from other objects, events, and actors” (2018, 13). By applying his perspective, networks can be understood in a way that allows for high levels of complexity and contingency. Furthermore, the process of such networks’ emergence and stabilization is of great interest to policy researchers in the sense that new networks of cooperation are developed to link elements that were previously disconnected (Jessop, 2011). Therefore, a crucial part of the theory behind the analysis of objects of governance is the notion that how objects of governance become defined as such is dependent on negotiation processes underlying sociotechnical controversies. A major quality of this approach is its capacity to explain how and why a specific version of an object of governance emerges. Such an analysis can be very useful because it helps provide new insights into the dynamic processes and (path-dependent) characteristics of technoscientific governance. Finally, the fact that this approach is very much open to novel, emergent understandings of the object of governance at hand can be quite useful. Instead of understanding robotic technologies as something pre-defined, the goal is to look at the way in which it is exactly the above-mentioned processes of interaction that are responsible for their constitution as an object of governance. The approach of analyzing new phenomena as objects of governance (or comparable concepts) is useful for social scientists because of its possibilities for applying a critical perspective: by developing an understanding of underlying governance processes, it becomes feasible to criticize their assumptions.

Nevertheless, there is a difference between the approach to objects of governance described above and the objective of this paper. The different studies mentioned above focus on (recent) pasts: they trace, often through qualitative empirical social research, how something emerges as an object of governance. This paper is neither focused on tracing the (recent) past of robotics governance, nor does it aim to systematically present the outcomes of empirical social research. Rather, it seeks to develop an understanding of robot standing as a speculative concept while conceptualizing its contribution to the process of robots becoming objects of future governance. In other words, the object of governance concept is used to exploratively establish the role of speculative concepts like robot standing in the governance of (social) robots, rather than descriptively criticizing existent and past robotics governance. As such, the paper focuses more strongly on the mission of philosophy of technology rather than the social sciences when it comes to these matters. In a more general sense, the argument here is that the systematic and robust application of speculative concepts can aid the process of constituting better, more profound objects of future governance that aid the process of implementing robots into our society in a sustainable manner. As previously stated, complex objects of governance by default go through different processes of negotiation along the lines of epistemic disagreements. Therefore, on a governance level, if philosophers (of technology) are provided with the possibility to engage with the development of policy ideas and demonstrate their insights, they can be participants in the negotiation processes behind sociotechnical controversies, with their concepts serving as their currency. In light of this, the section below will explain why and how robot standing can be seen as such a concept by framing the issue of robot standing as an important rhetorical and analytical device in the process of constituting robots as objects of governance.

## Robot Standing and the Governance of Social Robots

The preceding sections have explained how robot standing can be understood as a speculative concept that can aid the process of negotiating how (social) robots are to be constituted as objects of governance. The subsections below explore different uses of the robot standing concept in more detail. They describe the ramifications of applying the concept of robot moral standing in discussions on the futures of robots. In doing so, my aim is to develop some concrete insights and proposals of how a speculative concept like robot standing can be of help in the deliberative processes behind the development of new policy ideas. This should help to determine how some futures might be prevented so that other futures can be realized (Bratton, 2021). Three different points are distinguished: the understanding of social robots, analysis of robots’ societal impact, and the exploration of (social) robots’ sociotechnical potential.

### Facilitating New Understandings of Social Robots

Part and parcel of the analysis of the process in which objects of governance become constituted is the idea that specific policy ideas and the concepts related to them, are important for enabling governance in a volatile, high-stakes context (Schaper-Rinkel, 2013). However, from a governance standpoint, it is certainly impossible to track down every small-scale but potentially large-impact instance of technological development from the start and understand its consequences. What can be done is to develop different guiding concepts and narratives that are sufficiently broad while avoiding deterministic views of technological development. In such a context, the speculative endeavour towards concepts of moral standing can be described as attempts to provide more sophisticated understandings of social robot morality as such. Because of its disciplinary focus on the development of concepts and conceptual schemes, philosophy of technology plays an important role in developing those understandings. In recent decades, philosophy of technology and related fields have seen quite a transformation, which is often referred to as an “empirical turn” (Brey, 2010). Now that this turn has become quite established, the question is in which ways philosophy of technology should aim to influence policy ideas and improve the concepts that can be used in the negotiation processes behind the constitution of (social) robots as objects of governance. Since philosophers (of technology) have a great track record concerning the moral and mental standing of humans and other beings, it is desirable that they continue such activities. Whereas artificial concerns with no ground in engineering reality should probably be avoided, it is also important to actively learn what kind of speculative concepts have the ability to support the development of more sophisticated and profound understandings of robots as objects of governance. The question is therefore not whether we should have a concept of robot standing, but rather, what kind of concepts of robot standing we want to explore and which ones should better be set aside. Naturally, interdisciplinary and transdisciplinary interactions are crucial here in order to continuously discuss the (ir)relevance of specific concepts, tweak their definitions, and explore their potential ramifications.

When philosophers explore new ontologies and identify lacunae within existing ontologies, the goal is to create new understandings of the demarcation and definition of the meaning of robots in specific contexts. In this way, they can demonstrate the ways in which robots can disturb existing ontologies. Crucial here is that associated concepts can be applied in different contexts. Philosophical elaborations on such changes can in that way become relevant to many other academic disciplines, such as law, HRI, and critical governance studies. For example, in his abovementioned work on climate as an object of governance, Bentley Allan describes how the notion of the climate in governance shifted from a bioecological to a geophysical understanding, because “US state agencies drove billions of dollars into the institutions of knowledge production, altering their priorities, trajectories, and products” (Allan, 2017, 157). In the same way, social robotics is currently becoming defined via specific priorities, trajectories and products. In this regard, automation should not only be understood as the outcome of engineering inventions. It is also something that must be discovered in its context of development. Philosophers can help shape the debate around such phenomena so that they can be understood in new and better ways. As this paper has argued, the use of well-developed speculative concepts is instrumental in such forms of engagement. The role of the philosopher is thus not necessarily to speculate continuously. Instead, it is to engage with speculative concepts and apply philosophical rigour to their potential ramifications. Even though fully autonomous social robots are still far from being realized, it is important to engage with their technological potential in a rigorous manner so as to facilitate the new understandings of social robots and their roles within the social contexts in which they will play unprecedented roles.

### Enabling Critical Long-Term Analysis of Robots’ Societal Impact

The main use of the object of governance notion as an analytical tool is to capture how policy-making takes shape. This lens demonstrates how investing in speculative concepts can be instrumental for the constitution of new objects of governance. A major advantage of this is that the use of such concepts will make it possible to trace different societies’ views and narratives concerning those concepts over a longer period of time. Debates on robot standing, as a key example, will most certainly change considerably over time. Having this concept available renders it a possibility for social scientists to analyze the discourses and narratives around robot standing. Directly related to this, it is important to analyze what can be done to make the moral and sociopolitical assumptions behind robots as transparent as possible. From a governance perspective, it is therefore useful to look at robots as artificial social agents and establish in which ways the artificial sociality of robots can become defined. In this way, the analysis and decision-making processes concerning the impact of robots can become more pluralistic. For our analysis of the impact of robotics and other emerging technologies, we are currently still too often depending on analytical tools that have been criticized for years for their lack of nuance. For example, the effects of robotic technology on society and the economy in order to facilitate governmental decision-making are still mostly analyzed via quantitative, mostly macroeconomic indicators that measure the effect of robots and automation on a country’s GDP, its employment rate and so on (National Academies of Sciences, Engineering, and Medicine, 2017). Future-oriented concepts make it possible to analyze the effect of (social) robots in the long term in different (qualitative) ways, since changes in meaning can be traced with discursive methods, as demonstrated by the examples of research on objects of governance. The advent of social robots in an increasingly complex society full of contradictory regimes of information makes it important to improve this type of analysis.

Therefore, even though quantitative indicators will remain important, and rightly so, it is useful to aim for speculative concepts that are likely to remain relevant for a longer period of time and are based on both social and technical contexts. The choice of such concepts is not easy and will certainly include concepts and ideas that fade away later, as they will turn out to be unfit for how technological development actually comes about. Therefore, which concepts qualify as useful in this context and which do not will always be a point of discussion. This paper argues that robot standing can be seen as a useful concept because it engages with the potentialities of robotics while being clearly linked to both cultural fascinations and ethical and legal systems. Furthermore, qualitative and quantitative indicators can be used together to improve the analysis of how automated social robots can be implemented in social life. Such concepts are critical for engaging with the future, particularly for research in the social sciences and humanities. Once such concepts become established, it not only becomes possible to have informed discussions about potential characteristics of robots, but also to trace how such concepts develop in the long term. This allows social research to monitor and map the sociocultural notions regarding technologies like social robotics in a more credible and structural manner. In order to do that, we need concepts that can help to analyze a specific sociotechnical controversy in a rigorous manner (Marres, 2015). In this way, we will hopefully be able to improve our understanding of long-term dynamics in large-scale sociotechnical systems.

### Exploring Social Robotics’ Sociotechnical Potential

Finally, a concept like robot standing also allows for explorative imagination of the future as a way of motivating new, emancipatory social ontologies (Lewis et al., 2018). In this context, speculative explorations of robot moral standing can be used to analyze moral and legal adaptations to potential future characteristics of the social fabric. Generally speaking, the type of imaginative thinking that serves as a foundation for ideas for sociopolitical change has historically been an important element of ideas and concepts in the humanities and social sciences. For instance, in recent decades, posthumanist thinking has been an important field that has mobilized the technoscientific imagination in order to argue for new, more equal sociopolitical realities. Crucial to such contemplations of the posthuman being as a political subject are the fact that they do not need to reach the status of material reality. Important examples of such sociopolitical entities include the cyborg in Donna Haraway’s *A Cyborg Manifesto*, which was already mentioned in above. Another more recent recent example is Aaron Bastani’s *Fully Automated Luxury Communism* (Bastani, 2019). In what he explicitly calls a “manifesto”, Bastani calls for full automation and common ownership of that which is being automated. In certain ways, the discussion on robot rights and robot standing has already contributed to comparable issues. Two different examples can help to illustrate this, the first being the robot Sofia, which received citizen rights in Saudi Arabia, which in turn sparked several discussions on how a robot apparently has more rights in Saudi Arabia than other minorities. Another example closer to philosophy of technology comes from Kathleen Richardson, who presents a firm argument by claiming that many of the discussions concerning changing human-machine boundaries and associated calls for robot rights and standing merely appear to be progressive, while are in fact based on the old but persistent (Arestotelian) notions of humans as property. In her argument, granting rights to robots is synonymous to granting rights to slaves, which then serves as a way to ignore modern forms of human slavery in general (Richardson, 2015, 2016). Even though several of the arguments in this paper at least partly contradict Richardson’s ideas, it is important to appreciate the clarity and firmness of her arguments on anti-essentialism and its relation to the rejection of ontological differences between humans and machines. In this way, the powerful imagery of the social robot can lead to important discussions on human sociality.

Hence, I argue here that the social robot can be used as a point of sociopolitical reflection and imagination. This is certainly not a new argument. For instance, as Scott Selisker nicely describes in his study of the human automaton in American politics, such imagery of the automaton became a common trope in portrayals of totalitarian governance while also figuring as an important element in progressive accounts of future societies (Selisker, 2016). In the same way, with the help of imagery of potential technological developments, autonomous social robots can already be imagined as sociopolitical agents, even though they might never become actual reality. Looking at social robots as objects of future governance in this way means that current social ontologies are continuously scrutinized (Sætra, 2021). As our legal and ethical systems and values need to be critically reviewed in this process, powerful concepts like moral standing can be used as rhetorical devices that enable specific understandings of human vs robot moral standing in the negotiation space for values surrounding (social) robots. Rather than resistance against robots as such, ethical and judiciary concepts can be developed as robust and innovative instruments for debates that aim to create more equal futures with robots. Those utopian social ontologies can then be applied to criticize actual governance, particularly in light of ironic and subversive elements in their argumentation. Imagery of the posthuman other is often a simultaneously fascinating as well as daunting prospect. Nevertheless, from a governance perspective, it might be tempting to equate such efforts to the hubris that surrounds emerging technologies in general. In fact, in addition to the discussions on the potential effects of robotics on very fundamental habits, they stimulate and obligate important discussions on crucial concepts lying under the surface of society.

## Conclusion

Philosophy of technology has already made considerable efforts towards increasing involvement in the development of policy ideas. This paper has aimed to provide several arguments about how the speculative element of such efforts can be beneficial for the process of constituting social robots as objects of governance in an intelligent and informed manner. This paper has argued that the development of a concept like robot standing should be understood as an effort to develop concepts that are speculative but rigorous. Both are required to achieve this goal, which will also necessitate efforts to develop such concepts further while testing their usefulness outside of philosophy. With respect to the new normal of emerging technology, part of the solution can be found in the development of new idioms and imaginaries that can help to understand new technology and how its different futures (e.g. technological, social, political, economic) are incoherent with each other. Thus, it is important that speculative futures concerning emerging technologies be taken seriously and engaged with. Rather than understanding the technological future as a fantasmatic projection, the idea is to engage critically with it and its narratives. This also means that instead of disapproving of the future-grasping, speculative character of technological visions, there is a need to invest rather more than less into speculative concepts like robot standing. It is through the thorough analysis of these concepts that philosophy of technology can actively participate in the prescriptive engagement with technology futures.

## Data Availability

The original contributions presented in the study are included in the article/supplementary material, further inquiries can be directed to the corresponding author.
